# Integrating AI Literacy into Medical Education: Preparing Future Clinicians for an AI-Driven Healthcare System

**DOI:** 10.1007/s40670-025-02599-y

**Published:** 2026-01-05

**Authors:** Linda Chang, Radhika Sreedhar

**Affiliations:** 1 University of Illinois Chicago-Rockford College of Medicine, 1601 Parkview Ave Rockford, Il, Chicago, 61107 USA; 2 University of Illinois Chicago College of Medicine, 840 S. Wood Street Clinical Sciences North, Ste 440, Chicago, IL MC 718, 60612 USA

## Abstract

The rapid integration of artificial intelligence (AI) into healthcare necessitates curricular reforms to prepare future providers. Despite AI in medicine training frameworks from NAM and WHO, most medical students receive minimal AI education. Our institution developed a phased, theory-driven AI in Medicine curriculum guided by connectivism and constructivism. Using stakeholder engagement, needs assessment of 529 students, and backward design, we integrated AI literacy into preclinical, clinical clerkships, and an elective course. This spiral curriculum reinforces application through case studies and hands-on activities, fostering clinical relevance. Our implementation offers a scalable model for integrating AI competencies within the health professions curriculum.

## Background

The Food and Drug Administration (FDA) has cleared and approved over 1,200 AI medical algorithms in the United States as of August 2025. (1) Non-clinical AI programs that do not require FDA approval are also being deployed exponentially across healthcare settings in population health. They help identify and address gaps in health equity, revenue management, hospital care monitoring, and preventive care. In addition to having information readily available to assist physicians, AI systems can also help reduce diagnostic and therapeutic errors that are inevitable in human clinical practice. (2) As AI becomes increasingly embedded in healthcare systems, physician’s role will evolve toward accessing, assessing, interpreting, and applying that data-informed health information to patient care. (3) While future clinicians must develop competencies in data interpretation, probabilistic reasoning, and AI literacy, it is equally important to maintain and nurture the humanistic aspects of care—applying these tools with compassion, empathy, and clinical judgment. (4) Members of the healthcare team members do not need to know programming or the technical aspects of AI, but they have to understand its limitations of AI in healthcare and things that can cause error in the processes of algorithm creation/testing and implementation. They also need to know how these technologies impact the various domains of equity including accountability, fairness, validity, and relevance, such as major potential malfunctions like “dataset shift and data drift”. (5, 6).

A systematic review found that most of the 535 radiology-related studies regarding AI use were retrospective cohort studies with limited external validation and high potential for bias. (7) Similar results were reported by a study on AI in digital pathology, where 99% of studies had a risk of bias or external application concerns. (8) Adequate AI knowledge can help identify defective AI programs and prevent potential adverse impacts on patients, advancing the Quintuple Aim by improving patient outcomes and reducing overall healthcare costs. (9) The National Academy of Medicine (NAM) provides a framework supporting AI integration into medical curricula and The WHO provided recommendations with a focus on the ethics and governance of AI in health. (10, 11).

In this setting of rapid advancements in the field of AI and medicine, current medical training is inadequate to prepare the next generation of healthcare providers to practice in this new reality of AI in medicine. In response, organizations such as the NAM, WHO, and others provided AI in medicine competencies and advocated for innovative integration of AI training within the medical curriculum. (10–13) Although studies show that most medical students expect AI to shape their careers, the majority still report minimal to no formal education about AI during their training. (14–18) While this provides a clear direction for curriculum development, a major challenge persists: medical schools struggle to introduce new AI-related content amid already constrained curricular time and resources. A recent systematic review identified significant challenges including: (1) lack of faculty expertise; (2) limited awareness of available AI tools; (3) limited time and curriculum space infrastructure; (4) ethical concerns; (5) unclear benefit; and (6) balance traditional and innovative teaching methods). (19).

**Fig. 1:**
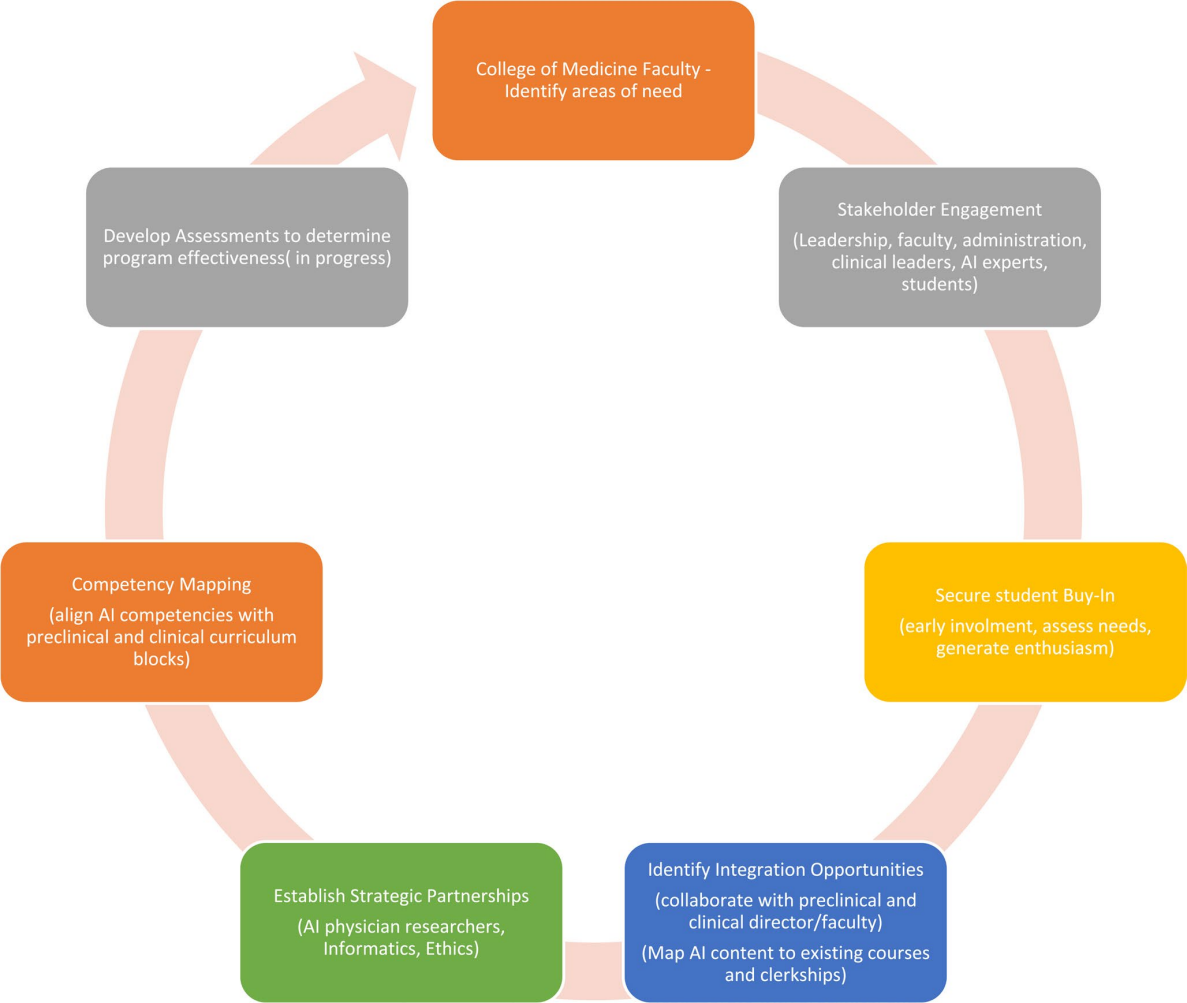
AI in medicine curriculum development: Phased approach flowchart

We initiated plans to design and implement an AI in Medicine curriculum following a deliberate, phased approach: engaging stakeholders, securing student buy-in, identifying opportunities for integration within existing courses, mapping relevant competencies to pre-clinical blocks and clinical clerkships, and establishing strategic partnerships to support implementation (Fig. 1). (10–13)

Our instructional design was guided by components of connectivism and constructivist learning theories. (20, 21) Connectivism supports an environment that students learn with experts from various disciplines, “networked learning environments” where learners connect to diverse teams of experts and learn to navigate information appropriately in this rapidly changing information related to AI models evolution in the healthcare settings. Constructivist theory emphasizes learning in context and creating a learning environment that is adaptive. It is a learner-centered approach where instructors provide a social environment for interactive learning, facilitating and guiding students through the learning process.

This curriculum stands apart because it uses live sessions that are integrated into the weekly content of the preclinical curriculum and aligned with clerkship specific content during the clinical years. These sessions demonstrate the real-world application of AI based tools, including their potential risks and benefits.

The primary goal of this paper is to share our development and implementation process with other medical educators, emphasizing lessons learned from our needs assessment, stakeholder engagement, and how we overcame the challenges encountered along the way. By sharing insights on how we incorporate AI literacy curriculum and the integration process, we aim to provide guidance and considerations that support other health educators in designing, adapting, and implementing similar AI curricula within their own institutions.

## Activity

Stakeholders were identified and their input was obtained. Faculty members expressed concerns regarding limited institutional resources, insufficient training opportunities, and the need for curriculum development support. We observed our medical students using AI despite having received formal training. In addition, a cross-sectional study was done to assess the learning needs, apprehensions, and digital self-efficacy of medical, nursing, and pharmacy students regarding the use of AI in healthcare. A total of 529 students participated, representing the colleges of medicine, nursing, and pharmacy. Across all disciplines, students strongly acknowledged the importance of learning about AI in healthcare, with high agreement rates for six core competency domains. Specifically, the student needs identified by the survey were AI applications (80%), evidence (82%), integration (85%), workflow (86%), positive impact (85%), and negative effects (92%). These findings reinforced the need to incorporate AI-related competencies into health professions education and informed the design of our phased implementation strategy. Following this, we worked closely with leadership and faculty from various disciplines to identify opportunities and develop content that is both relevant and feasible to integrate into existing curricular structures. As part of this process, we aligned AI-related content to established competencies within different organ system–based blocks and clinical/clerkship experiences, ensuring that integration was contextually meaningful and reinforced clinical relevance. (Tables 1 and 2).

**Table 1:** Content delivered in the preclinical and clinical years

Preclinical	Clinical
AI in Medicine: definitions and applications	AI in Family Medicine: AI and Clinical Workflows
Machine Learning Basics: supervised vs. unsupervised learning	AI in Neurology: AI and Data in Neurology.
Large Language Models	AI in Internal Medicine: AI and clinical decision support tools
How Visual AI works	AI in Obstetrics and Gynecology: Ambient AI in Obstetrics and Gynecology and ethical issues related
Statistical Basis of AI	AI in Psychiatry: Use of Social Media as a Source of Big Data in Psychiatry
Bias and Transparency in AI: how algorithms may perpetuate or mitigate disparities.	
Data sources, labelling and quality	
AI Ethics and Explainability	
Use of AI for Learning - strengths and pitfalls

**Table 2:** Mapping of competencies, learning objectives, to sessions and their delivery format

Competency and Specific Learning Objectives	Session Names and Delivery Format Superscript 1 = stand-alone live discussions Superscript 2 = discussions integrated into clinical session
1. Basic Knowledge of AI:	
A. Define artificial intelligence and its subfields and distinguish between machine learning and deep learning.	• Large Language Models Introduction^1^ • Diabetes core case- introduction to using AI for insulin pumps^2^
B. Differentiate between supervised, unsupervised, and reinforcement learning	• Diabetes core case- introduction to using AI for insulin pumps^2^
C. Discuss the importance of model interpretability and transparency in healthcare AI systems, especially for gaining trust from clinicians and patients.	• Strengths and pitfalls of using AI in research^1^ • Critical appraisal of an AI based study on predicting post operative acute kidney injury^1^
D. Discuss the importance of data quality, privacy, and security in healthcare.	• Introduction to cancer care core case: AI in pathology^2^ • Strengths and pitfalls of using AI in research^1^
E. Explain methods for preprocessing of medical data, and factors that influence the quality of data	• Introduction to cancer care core case: AI in pathology^2^ • Critical appraisal of an AI based study on predicting post operative acute kidney injury^1^
F. Describe the statistical basis of the outputs of AI -based applications.	• Dyspnea core case using AI for predicting readmission^2^ • Statistical basis of output of AI applications^1^
2. Evidence Based Evaluations of AI based Applications	
A. Demonstrate how ROC analysis, and precision-recall curves can be used in evaluating an AI model.	• Statistical basis of output of AI applications^1^ • Critical appraisal of an AI based study on predicting post operative acute kidney injury^1^
B. Assess if the dataset used in AI based application is reflective of the setting in which the model will be applied	• Critical appraisal of an AI based study on predicting post operative acute kidney injury^1^ • **Use of Social Media as a source of Big Data in Psychiatry- Psychiatry Clerkship**^**1**^
C. Appraise a dataset for potential biases and their implications in healthcare research involving AI-based applications.	• Leveraging AI for diagnosis - introduction to Visual Dx^2^ • Strengths and pitfalls of using AI in research^1^
D. Judge if the output of the AI based application is explainable.	• Strengths and pitfalls of using AI in research^1^
E. Assess if the AI based application’s performance is reproducible, and what metrics can be used in this assessment.	• Dyspnea core case - using AI for predicting readmission^2^ • Strengths and pitfalls of using AI in research^1^
3. Social and Ethical Implications of AI	
A. Analyze how data quality impacts the output of AI-based applications and can potentially reduce or exacerbate health disparities	• Diabetes core case- introduction to using AI for insulin pumps^2^ • Palpitations TBL -Automated EKG Reading pitfalls^2^ • **Use of Social Media as a source of Big Data in Psychiatry- Psychiatry Clerkship**^**1**^ • **AI and data – Neurology Clerkship**^**1**^
B. Evaluate potential issues regarding fairness and equity in the use of AI-based applications in health care.	• Strengths and pitfalls of using AI in research 1 • **Use of Social Media as a source of Big Data in Psychiatry- Psychiatry Clerkship**^**1**^
C. Reflect how personal and structural biases can impact health data and the outputs of AI-based applications.	• Strengths and pitfalls of using AI in research^1^ • Use of Social Media as a source of Big Data in Psychiatry^1^
D. Appraise ethical issues raised by various design, implementation, and use scenarios involving AI based applications.	• **Use of Social Media as a source of Big Data in Psychiatry- Psychiatry Clerkship**^**1**^ • **Ambient AI in Obstetrics and Gynecology and ethical issues related to them​-Obstetrics Clerkship**^**1**^ • Ethical Issues Related to the Use of AI Applications^1^
4. AI-Enhanced Clinical Encounters:	
A. Compare and contrast the indications for AI-based tools, vs. traditional methods.	• Large Language Models^1^
B. Judge when it is appropriate to apply AI-generated recommendations.	• Introduction to Evidence Based Medicine^2^ • Football injury core case- Clinical Decision Support System related to drug allergy^2^ • Large Language Models^1^ • Pharmacy Toxidromes^2^
C. Explain to patients the risk and uncertainty as they relate to the outputs of AI-based applications and describe practical implications for their care.	• **AI based Point of Care tools for Internal Medicine​ Internal Medicine Clerkship**^**1**^ • **AI and Sepsis Prediction– Internal Medicine Sub internships**^**1**^
D. Demonstrate(by reflection) comfort and humility in caring for data-empowered patients and incorporate patient-reported data and outcomes in developing care plans.	• Ethical Issues Related to the Use of AI Applications^1^
E. Propose how AI-based applications can be used to enhance access and quality of care in remote and underserved settings.	• Large Language Models^1^ • AI in predictive modeling for disease outbreaks^2^
5. Workflow Analysis for AI-Based Applications	
A. Formulate collaborative team-based approaches to potential changes in roles, responsibilities, and workflows associated with the adoption of novel AI-based applications	• Pharmacology learning module-drug discovery^2^ • **AI and clinical workflows clinic messages from patients- Family Medicine Clerkship**^**1**^ • **Ambient AI in Obstetrics and Gynecology and ethical issues related to them​-Obstetrics Clerkship**^**1**^
6. Practice-Based Learning and Improvement Regarding AI-Based Applications	
A. Discuss medicolegal and regulatory frameworks governing the development, deployment, (e.g., FDA approval process) of AI for clinical uses including practice-based improvement activities	• Pharmacology learning module-drug discovery^2^

**Sessions in the clerkship are bolded

**Competencies adapted and modified from reference #12,13

Our approach applied components of connectivism and constructivist learning theories and backwards design principles to develop learning assignments that simulate the real-world work done in the healthcare industry. Content was created and delivered by a multidisciplinary team across three campuses. Core faculty led the review process and provided faculty training prior to session delivery. This student-centered course is designed to teach critical divergent thinking and active learning – not just “knowing,” but also “doing.” This involved classroom team activities with hands-on application activities in the domains of discovery, preparation, analysis, processing, and explaining the clinically relevant medical information to patients. By aligning our initiative with these complementary theories, we aim to foster both a foundational understanding of AI literacy and evaluation and the application of emerging AI technologies within clinical practice.

## Results

Guided by the learning theories and backward design, we developed a longitudinal, integrated spiral AI in Medicine curriculum. This spiral curriculum implemented in fall 2024 has three components and aims to prepare students to manage patients in the era of AI- technology augmented healthcare settings. Competencies and learning objectives were adapted from the expert consensus that identified six AI competency domains (Table 2). The first component is in the preclinical curriculum where AI-literacy concepts were integrated and introduced in the foundational unit, with content progressively expanding in subsequent organ systems. Application-based learning was reinforced through short case studies integrated into the cardiovascular, nephrology, and Behavior organ systems. The second component, housed in the clinical years, engages students with various AI in Medicine tools, emphasizing complex case-solving activities that incorporate AI technology within diverse clerkship settings. The focus here is on the understanding of the impact of AI-technology on healthcare delivery and its roles in augmenting patient care. Spiraling was achieved by revisiting the learning objectives in either the 50 min stand alone or 15 to 20 min integrated session. (Table 2). These components were delivered to 300 students in each year of medical school. The third component is a concentrated, month-long elective for senior medical students. This modular, four-week course integrates AI with evidence-based medicine, pathology, pharmacology, telemonitoring, quality improvement, value-based care, patient safety, clinical reasoning, communication, and patient care skills. The elective structure, assessment methods, and outcomes have been previously published. (22).

Our course evaluation incorporates the Kirkpatrick model and applying the first 3 levels of assessment. (23) We have implemented assessments to capture learners’ engagement and satisfaction through end-of-session surveys and short written reflections reflecting reaction. Assessments are also in place to assess knowledge using multiple-choice questions, and checklists during simulation sessions to assess knowledge. Behavior Level assessments that have been implemented include faculty grading checklist in simulations and team-based activities, and reflective write-ups on applying AI tools in clinical scenarios.

## Discussion

We demonstrate the development and implementation of an AI in Medicine curriculum in medical school. The lessons learned from needs assessment, stakeholder engagement, and challenges encountered in implementation would be of value to the readers who wish to develop and implement a similar curriculum. This curriculum design reflects the core concepts of connectivism, constructivist learning theories by emphasizing active knowledge construction, contextual learning, and learner engagement. Foundational content and case-based discussions in the preclinical years lay the groundwork, while an hour-long discussion session on clinical cases in clerkships allowed students to apply what they had learned and discuss their understanding in practice-based settings. The time constraints were overcome by thoughtful alignment and integration of concepts in preexisting curricular content where feasible. Stakeholder engagement was key in identifying partners who were willing to deliver content across three campuses.

While this curriculum represents a thoughtfully designed approach to integrating AI competencies within an existing full medical curriculum, it is not without limitations. As the curriculum is in its implementation phase, evaluation of its effectiveness in achieving the intended learning outcomes is limited as students have not completed the entire curriculum. Preliminary results from our multiple-choice assessments indicate a positive trend in students’ acquisition of AI knowledge. Additional summative evaluation and program-level metrics are underway.

## Conclusion

This proposal presents a theoretical and strategic framework grounded in educational theory and informed by a comprehensive needs assessment. However, the lack of outcome-based evaluation at this early stage limits our ability to determine the extent to which the curriculum fosters measurable improvements in AI literacy, critical thinking, or clinical application. We also recognize that AI curricular integration is highly context-dependent, as there are differences in institutional curriculum structures. Nonetheless, the integrated, spiral, competency-based AI curriculum described here offers a model that can be adapted by other institutions seeking to prepare future physicians to care for patients in an evolving, AI-enabled healthcare setting.
